# Variations of Gut Microbiome Profile Under Different Storage Conditions and Preservation Periods: A Multi-Dimensional Evaluation

**DOI:** 10.3389/fmicb.2020.00972

**Published:** 2020-05-27

**Authors:** Junli Ma, Lili Sheng, Ying Hong, Chuchu Xi, Yu Gu, Ningning Zheng, Mengci Li, Linlin Chen, Gaosong Wu, Yue Li, Juan Yan, Ruiting Han, Bingbing Li, Huihui Qiu, Jing Zhong, Wei Jia, Houkai Li

**Affiliations:** ^1^Functional Metabolomic and Gut Microbiome Laboratory, Institute of Interdisciplinary Integrative Medicine Research, Shanghai University of Traditional Chinese Medicine, Shanghai, China; ^2^Shanghai Key Laboratory of Diabetes Mellitus, Center for Translational Medicine, Shanghai Jiao Tong University Affiliated Sixth People’s Hospital, Shanghai, China; ^3^Department of Endocrinology, The Fifth People’s Hospital of Shanghai, Fudan University, Shanghai, China; ^4^Huzhou Key Laboratory of Molecular Medicine, Huzhou Central Hospital, Affiliated Central Hospital Huzhou University, Huzhou, China; ^5^University of Hawaii Cancer Center, University of Hawai‘i at Manoa, Honolulu, HI, United States

**Keywords:** fecal samples, storage conditions, storage periods, sequencing platform, microbial profile

## Abstract

Gut dysbiosis is heavily involved in the development of various human diseases. There are thousands of publications per year for investigating the role of gut microbiota in diseases. However, emerging evidence has indicated the frequent data inconsistency between different studies, which is largely overlooked. There are many factors that can cause data variation and inconsistency during the process of microbiota study, in particular, sample storage conditions and sequencing process. Here, we systemically evaluated the impacts of six fecal sample storage conditions (three non-commercial storage protocols, −80°C, −80°C with 70% ethanol (ET_−80°C), 4°C with 70% ethanol (ET_4°C), and three commercial storage reagents, OMNIgeneGUT OMR-200 (GT) and MGIEasy (MGIE) at room temperature, and Longsee at 4°C (LS) on gut microbiome profile based on 16S rRNA gene sequencing. In addition, we also investigated the impacts of storage periods (1 and 2 weeks, or 6 months) and sequencing platform on microbiome profile. The efficacy of storage conditions was evaluated by DNA yield and quality, α and β diversity, relative abundance of the dominant and functional bacteria associated with short-chain fatty acid (SCFA) production, and BAs metabolism. Our current study suggested that −80°C was acceptable for fecal sample storage, and the addition of 70% ethanol had some benefits in maintaining the microbial community structure. Meanwhile, we found that samples in ET_4°C and GT reagents were comparable, both of them introduced some biases in α or β diversity, and the relative abundance of functional bacteria. Samples stored in MGIE reagent resulted in the least variation, whereas the most obvious variations were introduced by LS reagents. In addition, our results indicated that variations caused by storage condition were larger than that of storage time and sequencing platform. Collectively, our study provided a multi-dimensional evaluation on the impacts of storage conditions, storage time periods, and sequencing platform on gut microbial profile.

## Introduction

The mammalian gastrointestinal tract is the main site for commensal bacteria, which contains at least 1.3 times as many genes as host genome (Savage, 1977; Jia et al., 2008; Nicholson et al., 2012; Sender et al., 2016). In recent years, the passion on gut microbiota-related research is overwhelming due to the involvement of gut dysbiosis in development of various human diseases including obesity, diabetes mellitus, non-alcoholic fatty liver diseases, cardiovascular disease, and even cancers (Abu-Shanab and Quigley, 2010; Jia et al., 2018; Ma and Li, 2018; Canfora et al., 2019). The advances of high-throughput sequencing technologies including 16S rRNA gene and metagenomics lay the solid foundation for investigating the role of gut microbiota in human diseases (Liu et al., 2017; Wu et al., 2019).

There are thousands of microbial-related publications per year mainly by using 16S rRNA gene sequencing technology. However, it should be noted that gut microbiome data are usually variable and not very consistent between different studies (Wu et al., 2011; Yatsunenko et al., 2012; Walters et al., 2014). There are many ways for introducing disturbance on the diversity or composition of microbiome during the whole experimental process including sample collection, transportation, storage, DNA extraction, sequencing, and biometric analysis (Costea et al., 2017; Penington et al., 2018; Schloss, 2018). Data derived from 103 fecal samples of two infant cohorts show that fecal microbial structure changes significantly during ambient temperature storage after 2 days, so immediate freezing at −80°C or DNA extracted within 2 days is suggested if samples were stored at room temperature (Shaw et al., 2016). However, another study reveals that fecal samples stored at room temperature beyond 15 min would result in obvious variation in bacterial taxa, whereas even the usage of microbial nucleic acid stabilizer RNAlater also caused dramatic reduction in DNA yields and bacterial taxa (Gorzelak et al., 2015). Generally, immediate freezing at −80°C is supposed to be the golden standard for most biological samples including feces for microbiome study (Fouhy et al., 2015; Al et al., 2018). However, obvious alteration in microbial community is also observed in samples frozen at −80°C compared to that of fresh samples, suggesting that −80°C might not be the most optimal method for fecal sample storage (Horng et al., 2018). Moreover, immediate freezing at −80°C is usually unfeasible in many cases for field studies, or at the circumstance where the fecal samples are collected at home by patients themselves. In these cases, samples are apt to be exposed at ambient temperature during collection and transportation before DNA extraction. Currently, various commercial and experimental preservation reagents for fecal sample have been developed or trialed such as the OMNIgene Gut kit, RNAlater, FTA cards, 70% or 95% ethanol, 50:50 glycerol:PBS, NOBP-based reagent, and so on (Flores et al., 2015; Song et al., 2016; Han et al., 2018). Although some comparisons between these preservation reagents have been performed, inconsistent or even contradictory results are also observed. The inconsistent results might be associated with the differences in sample donors, definitions for the “Fresh” control samples, DNA extraction protocol, data processing, and so on. Song et al. (2016) find that the preserving effect of 95% ethanol is comparable with that of FTA cards or OMNIgene Gut kit at ambient temperature, but strongly caution against the use of 70% ethanol for fecal sample preservation. In contrast, Horng et al. (2018) report that the microbial composition of canine samples preserved with 70% ethanol or RNAlater closely resembles that of fresh samples, and they suggest that 70% ethanol is the best method for fecal sample preservation. Contradictory results are also observed in the use of RNAlater. One study shows that fecal samples preserved with RNAlater closely resemble fresh samples (Horng et al., 2018), whereas the least similarity in microbial composition and abundance with fresh samples are reported in another study (Hale et al., 2015). In addition, significant differences are also observed in microbial profile of identical samples that are processed and sequenced at two research centers (Sinha et al., 2016; Penington et al., 2018). The Microbiome Quality Control (MBQC) project baseline study (MBQC-base) reports that each microbiome protocol step, including sample handling environment, DNA extraction, and bioinformatic processing, has the potential to introduce variation of comparable effect size to that of biological differences (Sinha et al., 2017), thus, it is of vital significance to systemically evaluate the variations of gut microbiome data that could be introduced during the process of sample preparation such as different fecal storage conditions, period of preservation, and even different sequencing platforms.

**FIGURE 1:**
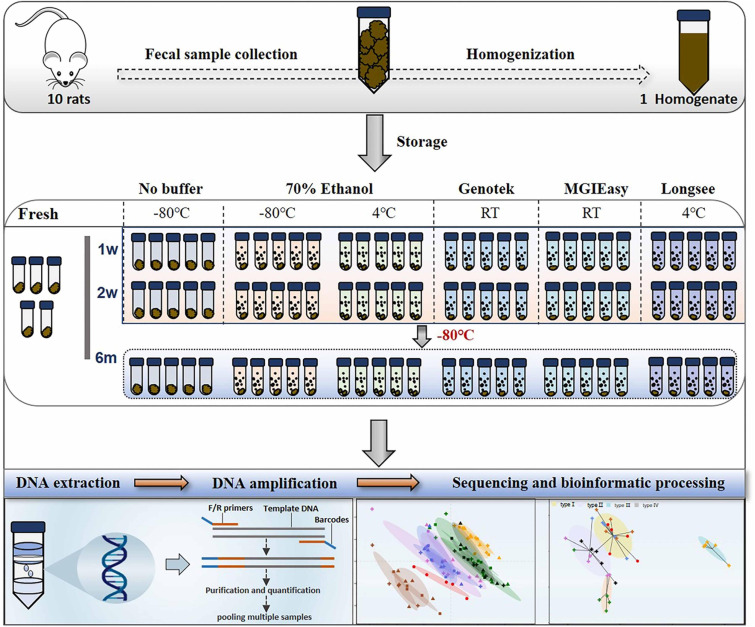
Diagram of experimental design. Fecal samples from 10 rats were quickly collected into sterile 50 ml tubes, homogenized, and then aliquoted. Next, these aliquots were treated with a range of preservatives. Bacterial DNA were extracted at 4 time points: on the day of sampling (“Fresh”) as well as after 1 week, 2 weeks, and 6 months of storage, followed by DNA extraction and 16S rRNA gene sequencing.

In the current study, we systemically evaluated the extent of variations of gut microbiome profile introduced by different storage conditions. We collected the fresh fecal samples from rats and pooled the samples for homogenization. Then, the homogenized sample was allocated into various replicates for observing the impacts of 3 commonly used storage conditions, −80°C, addition of 70% ethanol at either −80°C (ET_−80°C) or 4°C (ET_4°C), and 3 commercial stabilizers including OMNIgeneGUT OMR-200 (GT) and MGIEasy (MGIE) at ambient temperature, and Longsee (LS) at 4°C according to their instructions. Bacterial genomic DNA was extracted with same protocol at the end of the 1st week, 2nd week, and 6th month, respectively, as well as the fresh samples. The study design is shown in Figure 1. All of the samples were subjected for gut microbiome profiling by using 16S rRNA gene sequencing. Our results demonstrated that fecal storage conditions did have dramatic impacts on gut microbiome profile including DNA quality, α or β diversity, relative abundance of some functional bacteria in SCFA production and bile acid metabolism. We confirmed that −80°C was acceptable for fecal sample storage, and the addition of 70% ethanol was beneficial for maintaining the original community composition of the dominant phyla. Meanwhile, we found that gut microbiome profiles of samples stored in ET_4°C and GT reagents were similar regarding to their impacts on community α or β diversity, and the relative abundance of dominant and functional bacteria. Our results showed that gut microbiome profile of samples stored in MGIE reagents was the closest to that of fresh samples, while LS reagents introduced the most obvious data variation. Finally, we also confirmed that variations caused by ways of storage condition were larger than that of storage periods and sequencing platform.

## Results

### Do Different Storage Conditions Lead to Variation in DNA Yield or Quality?

**FIGURE 2:**
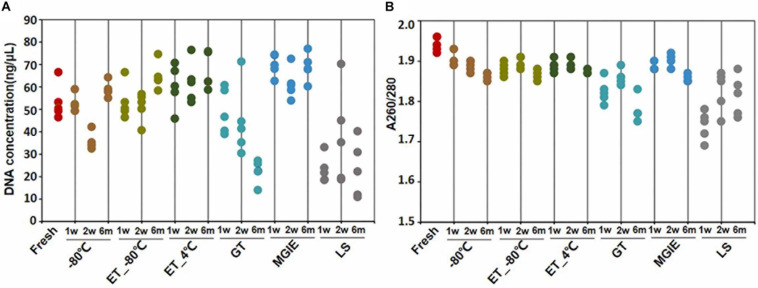
DNA quality under different storage conditions. **(A,B)** The concentration and purity of DNA extracted from fecal samples under different storage conditions.

DNA quality is the basis for microbial study. However, previous study indicates that DNA quality is affected by DNA extraction protocols (Costea et al., 2017). To determine the impact of fecal storage conditions on bacterial genomic DNA quality, we evaluated the concentration and purity of DNA extracted with same protocol from samples under different storage conditions. Despite obvious fluctuations, our results showed that DNA concentrations were comparable among most samples, except for the relatively higher in MGIE and lower DNA concentrations in GT and LS reagents at the 3 time points of storage (Figure 2A). Meanwhile, DNA quality was evaluated with the absorption ratio of 260/280 nm. We found that most samples showed satisfactory value in 260/280 from 1.8 to 2.0, except for samples stored in LS reagents with relatively lower 260/280 value. Notably, DNA concentration of samples stored in GT reagents showed obvious reduction with time (Figure 2B). Taken together, our current data suggested that most storage conditions had minor and acceptable impacts on DNA yields or quality, whereas samples in LS reagents showed relatively lower yields and quality. Preservation of samples in GT reagents might result in reduction of DNA yield time-dependently.

### Do Different Storage Conditions Affect Bacterial Diversity?

**FIGURE 3:**
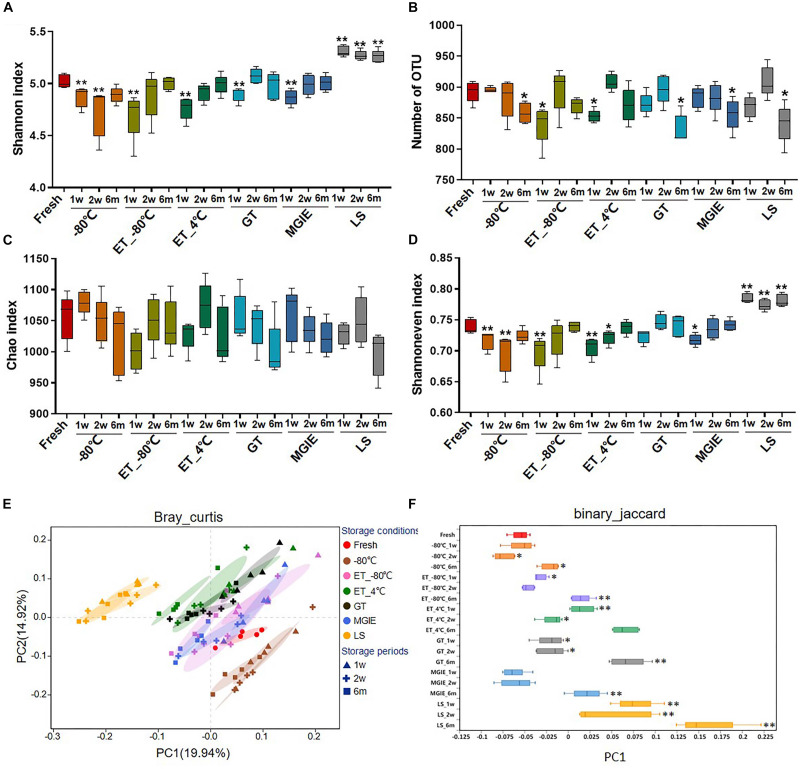
The effects of preservation methods on community diversity. **(A)** Community α diversity was measured by Shannon diversity index. **(B)** Number of OTU. Community richness and evenness were evaluated by **(C)** Chao1 estimator and **(D)** Shannon index-based measure of evenness. Community β diversity was measured by PCoA plots based on **(E)** Bray–Curtis and **(F)** the discrete distribution of β diversity on PC1 based on binary_jaccard. *n* = 5 per group. ^∗^*p* < 0.05, ^∗∗^*p* < 0.01, compared with fresh group.

Bacterial diversity is the main character for investigating the role of gut microbiota in disease. In the current study, a total of 5,645,994 sequences were obtained from 95 samples, and the summary table with sequencing information was provided in Supplementary Table S1. After normalization, a total of 31,806 sequences for each sample were kept, and used for subsequent analysis. The Rarefaction and Shannon curves suggested that the current sequencing depth was sufficient for covering the majority of bacterial diversity in each sample (Supplementary Figures S1A,B). First, we compared the bacterial α diversity based on the Shannon and Simpson indices, number of OTUs, as well as Chao1 and Shannon evenness estimators under different storage conditions. Our results showed that the bacterial α diversity was differently altered in samples stored at −80°C for 1 or 2 weeks, and 6 months, ET_−80°C and ET_4°C for 1 week, as well as GT and MGIE reagents for 1 week, characterizing as significantly varied Shannon and Simpson diversity indices, and number of OTU compared with fresh samples (Figures 3A,B and Supplementary Figure S1C), suggesting significant variation in diversity of either richness or evenness. Meanwhile, the comparable Chao index suggested that no significant differences in bacterial richness among different storage conditions, but considerable variation in evenness as shown by Shannon even index (Figures 3C,D). These data suggested that preservation conditions mainly affected evenness, but not richness of microbial diversity. Notably, samples in LS reagents showed relatively poor consistency in microbial α diversity with fresh samples (Figures 3A–D).

Next, Principal Coordinate Analysis (PCoA) based on binary_jaccard and Bray–Curtis were performed, which showed obvious variation in microbial community under different storage conditions. Generally, samples from the same storage condition for different periods were clustered together compared to storage conditions, suggesting that impacts of storage conditions are larger than storage periods. Specifically, samples stored at −80°C, ET_−80°C, and MGIE reagents were clustered closely to fresh samples, and followed by those in GT or ET_4°C, while samples in LS showed the worst clustering with fresh samples (Figure 3E and Supplementary Figures S2A,B), which was consistent with discrete distribution of β diversity on PC1 (Figure 3F). Collectively, these results suggested that storage conditions had dramatic impact on α or β diversity of gut microbiota to different extent compared with their fresh control, and the impact of storage condition superseded that of storage periods.

### How Do Different Storage Conditions Affect the Abundance of Dominant Bacteria?

**FIGURE 4:**
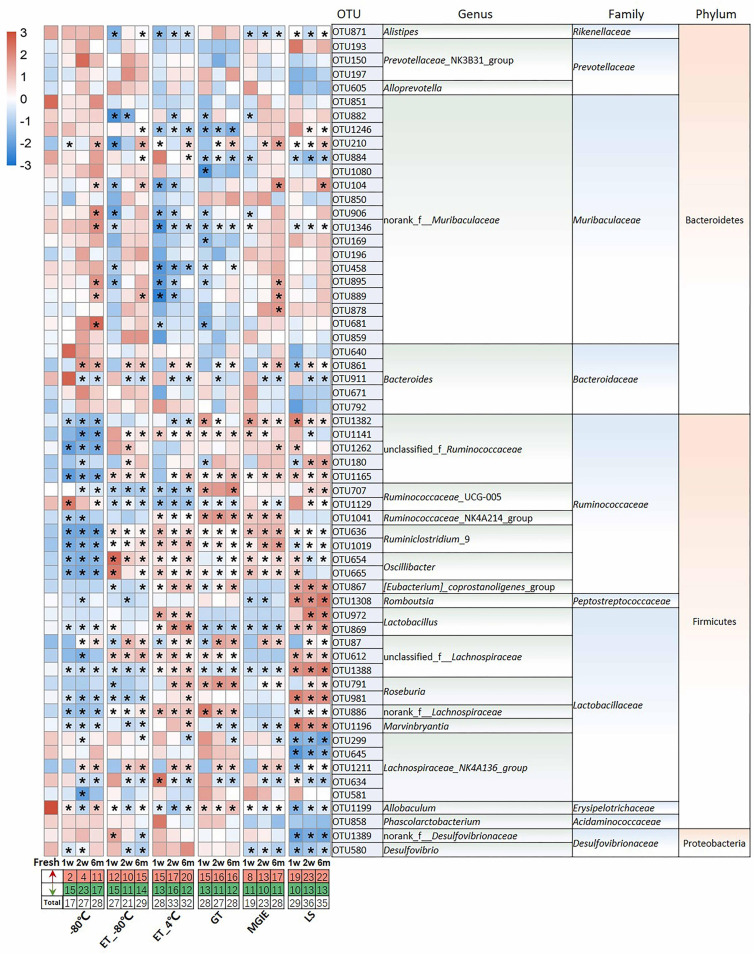
The impact of preservation methods on the top 60 OTUs. Heatmaps shown was the differences in relative abundance among groups of the top 60 OTUs, which accounted for about 65% of coverage, as well as their taxa information including genus, family, and phylum. The red and green entries indicate the number of OTU that were significantly more or less abundant under different storage conditions relative to fresh samples, respectively. *n* = 5 per group. *p < 0.05 and FDR < 0.05, compared with fresh group.

Given the observed impacts of storage conditions on bacterial α or β diversity, we further investigated the variations in the dominant bacteria by comparing the top 60 OTUs (30, 28, 2 from Firmicutes, Bacteroidetes, and Proteobacteria, respectively) accounting for about 65% of coverage, which were ranked by comparing the average abundance of each OTU across all samples. As shown in Figure 4, we found that different storage conditions resulted in dramatic changes to different extent in abundance of most OTUs compared to fresh samples. By contrast, we found that the numbers of altered OTUs in samples at −80°C, ET_−80°C, and MGIE reagents were relatively smaller than those in ET_4°C, GT or LS reagents. Interestingly, the majority of altered OTUs in samples stored at −80°C showed decreased abundance, while addition of 70% ethanol could balance the ratio of up- or down-regulated bacteria at −80°C. It was also notable that the OTUs abundance under different storage conditions were changed either time-dependent or –independently. For example, the samples stored at −80°C, ET_4°C and MGIE resulted in time-dependent increase in number of up-regulated bacteria, however, the number of down-regulated bacteria was random under most conditions. In addition, much more universal alteration was observed in OTUs belonging to Firmicutes phylum under different storage conditions than that of Bacteroidetes (Figure 4).

**FIGURE 5:**
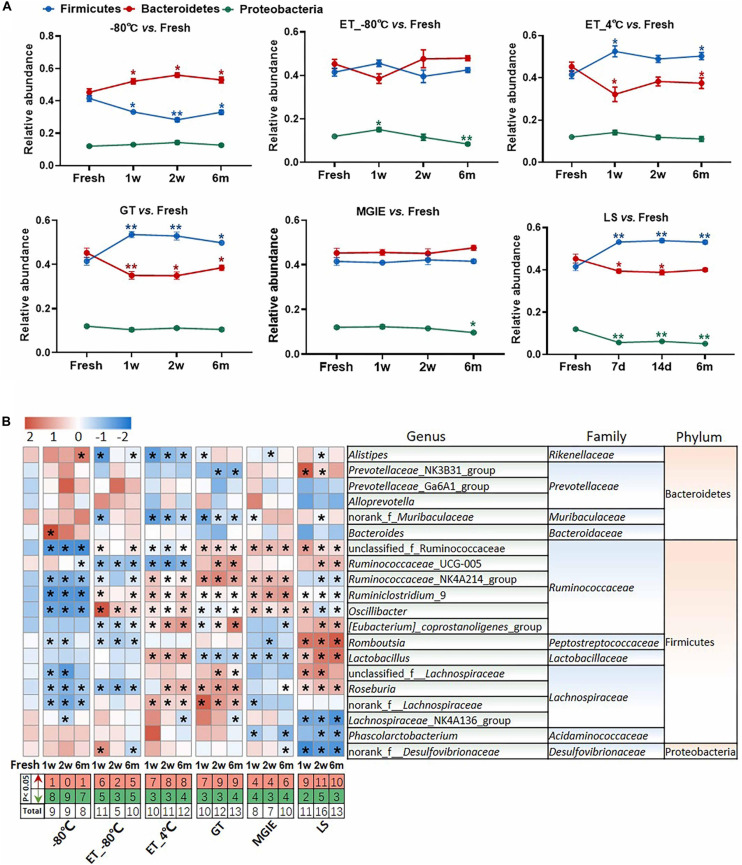
The effect of preservation methods on the dominant bacteria. **(A)** The effect of storage conditions on the relative abundance of the dominant phyla. **(B)** The heatmap shown was the differences in relative abundance among groups of the top 20 genera, which accounted for about 85% of coverage, as well as their taxa information including family and phylum. The red and green entries indicate the number of genus that were significantly more or less abundant under different storage conditions relative to fresh samples, respectively. *n* = 5 per group. *p < 0.05, **p < 0.01, compared with fresh group.

We next evaluated the change in relative abundance of the 3 dominant phyla, Firmicutes, Bacteroidetes, and Proteobacteria, as well as genera under different storage conditions by comparing with fresh controls individually. In comparison with fresh controls, −80°C storage resulted in obvious increase in Bacteroides and decrease in Firmicutes time-dependently, but not Proteobacteria. Interestingly, addition of 70% ethanol at −80°C (ET_−80°C), but not 4°C (ET_4°C) storage showed benefit for keeping the abundance of both Firmicutes and Bacteroidetes close to fresh control at the 3 time points, except for Proteobacteria. Samples stored in either GT or LS reagents resulted in obvious increase in Firmicutes and decrease in Bacteroidetes time-dependently, as well as decreased abundance of Proteobacteria only in LS reagents. Notably, the relative abundance of the 3 dominant phyla was well maintained at the 3 time-points in MGIE reagents storage compared to fresh control (Figure 5A).

The imbalanced ratio of Firmicutes to Bacteroidetes (*F*/*B*) was frequently observed in many diseases (Ley et al., 2006; Turnbaugh et al., 2006; Mouzaki et al., 2013; Zhu et al., 2013), therefore, the evaluation of *F*/*B* ratio is of significance for investigating the role of gut microbiota in disease development or drug efficacy. Our results showed that *F*/*B* ratios of samples at ET_−80°C and MGIE reagents were of little difference to that of fresh samples, whereas increased *F*/*B* ratios were observed in ET_4°C, GT and LS reagents, but decreased at −80°C (Supplementary Figure S3A). Consistent with previous results at phylum level, further analysis of the top 20 genera (85% of coverage) showed that samples at −80°C, ET_−80°C, and MGIE reagents introduced the minimum biases, followed by ET_4°C and GT reagents, whereas LS reagents caused the most obvious variation with up to 40 altered genera in total. Interestingly, we found that the majority of altered genera at −80°C were decreased abundance at 3 time points, while addition of 70% ethanol at −80°C balanced the number of increased and decreased genera. On contrary, alterations introduced by ET_4°C, GT and LS reagents were mainly increased. It was found that most of the variable genera were belonging to Gram-positive *Ruminococcaceae*, such as *Ruminococcaceae*_UCG-005, *Ruminiclostridium*_9, and *Oscillibacter*, whereas bacteria in genera of *Prevotellaceae_*NK3B31_group, *Alloprevotella* and *Bacteroides* were of minor change under different conditions belonging to Gram-negative *Prevotellaceae*, and *Bacteroidaceae*. The thicker cell wall of Gram-positive cells makes them more resistant to mechanical forces, which may account for the higher variability than Gram-negative bacteria. Nevertheless, we did not observe the time-dependent impacts of storage periods on number of altered genera under all of the storage conditions (Figure 5B). In addition, correlation analysis between observed conditions and fresh samples was performed with Spearman’s correlation coefficient (SCC). Our results showed that samples at −80°C and ET_−80°C had the highest correlation with fresh samples ranging from 0.85 to 0.9, followed by ET_4°C, GT, and MGIE reagents ranging from 0.75 to 0.85. Samples stored in LS reagents exhibited the worst similarity to fresh samples, in which the SCC was less than 0.7 (Supplementary Figure S3B). Collectively, our results indicated that samples at ET_−80°C and MGIE reagents kept the dominant phyla relatively stable including Firmicutes, Bacteroidetes, and Proteobacteria at 3 time points. Samples in LS reagents showed relatively higher variations at all levels. Moreover, the impacts of short- or long-term storage were not very significant under the same condition.

### Do Different Storage Conditions Alter the Relative Abundance of Functional Bacteria?

**FIGURE 6:**
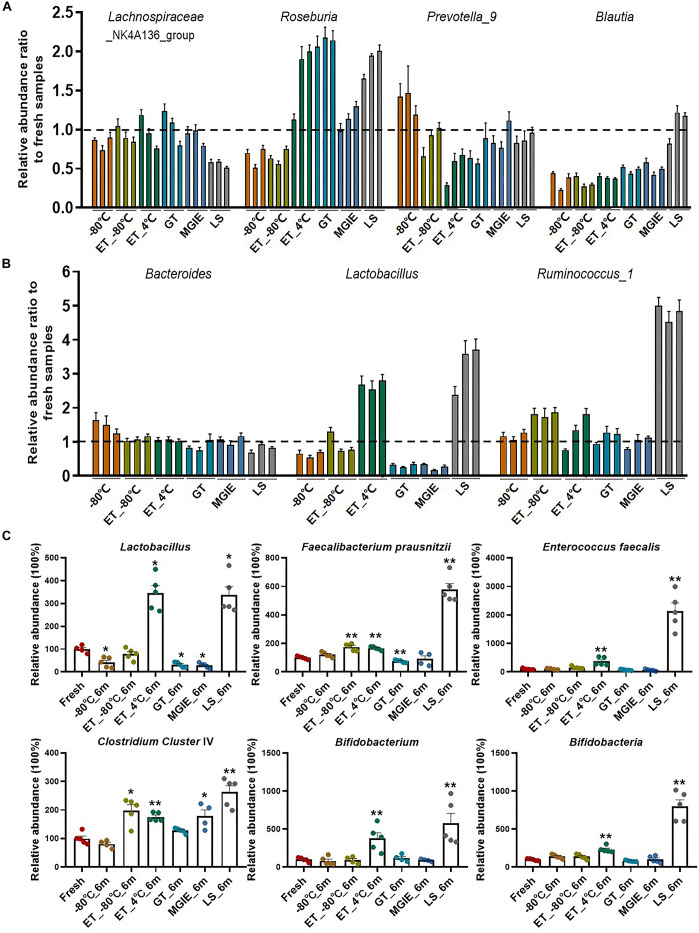
Relative abundance ratio of bacteria involved in SCFA-producing and bile acid metabolism on genus levels. **(A,B)** Relative abundance ratio (storage samples/fresh samples) of bacteria involved in SCFA-producing and bile acid metabolism on genus levels, and three columns with the same color represent 1 week, 2 weeks, and 6 months from left to right, respectively. **(C)** Qualification of the functional bacteria relative to fresh samples using QPCR. *n* = 5 per group. *p < 0.05, **p < 0.01, compared with fresh group.

Increasing evidence has confirmed that gut microbiota play critical roles in maintaining human health or disease development by producing microbial metabolites like bile acids or SCFAs that serve as signaling molecules or energy substrates (Jia et al., 2018), however, few attentions have been paid to the influences of storage conditions on change of functional bacteria yet. Therefore, we evaluated the relative abundance ratio (stored samples/fresh samples) of bacteria at genus level at 3 time points that are involved in SCFA-production including genera of *Lachnospiraceae*_NK4A136_group, *Roseburia*, *Prevotella*_9, and *Blautia*, as well as those in both SCFA-producing and bile acid metabolism including *Bacteroides*, *Lactobacillus*, and *Ruminococcus*_1 (Koh et al., 2016; Brown and Hazen, 2018). First of all, we found that even −80°C storage caused some changes of these bacteria at genus level such as obvious reduction of *Lachnospiraceae*_NK4A136_group, *Roseburia* and *Blautia*, and increasing of *Prevotella*_9 and *Bacteroides*. Addition of 70% ethanol at −80°C produced similar effect with −80°C, except for some benefits in *Prevotella_9*, *Bacteroides*, and *Lachnospiraceae_*NK4A136_group which were closer to fresh samples. However, storage at ET_4°C caused dramatic variations in most of these bacteria at a time-dependent way, except for *Bacteroides.* There were also obvious variations in abundance ratio of these bacteria in the 3 commercial stabilizers. Interestingly, the change trends were much more similar between GT and MGIE such as bacteria in *Blautia*, *Lactobacillus*, and *Ruminococcus*_1, while samples stored in LS reagents showed dramatic differences in majority of the observed bacteria, especially the unique change in *Lachnospiraceae*_NK4A136_group, *Blautia*, *Lactobacillus*, and *Ruminococcus*_1 (Figures 6A,B). We also found that *Bacteroides* and *Lachnospiraceae*_NK4A136_group were relatively stable under these storage conditions. Thus, our data suggested that different storage conditions could cause diversified fluctuations in the relative abundance of functional bacteria in a time-dependent or –independent way.

In addition, to further confirm the effect of storage conditions on the functional bacteria, we qualified the abundance of bacteria involved in bile acids metabolism and SCFAs metabolism (*Lactobacillus*, *Faecalibacterium prausnitzii*, *Enterococcus faecalis*, *Clostridium Cluster* IV, *Bifidobacterium*, and *Bifidobacteria*) with qPCR in samples stored for 6 months. We found that different storage conditions resulted in dramatic alterations to different extent in abundance of most bacteria compared to fresh samples. Detailly, −80°C storage caused alterations in *Lactobacillus*, *F. prausnitzii*, and *Bifidobacteria*. Addition of 70% ethanol at −80°C produced similar effect with −80°C in *F. prausnitzii* and *Bifidobacteria*, and provided the benefit for *Lactobacillus*, while caused increasing of *E. faecalis* and *Clostridium Cluster* IV. Except for *F. prausnitzii* and *Bifidobacteria*, samples in GT and MGIE reagents introduced comparable variations in *Lactobacillus* and *Clostridium Cluster* IV. Samples storage at ET_4°C and in LS reagents resulted in dramatic increase in all of the tested bacteria (Figure 6C).

### Do Different Sequencing Platforms Generate Consistent Results of Identical Samples?

**FIGURE 7:**
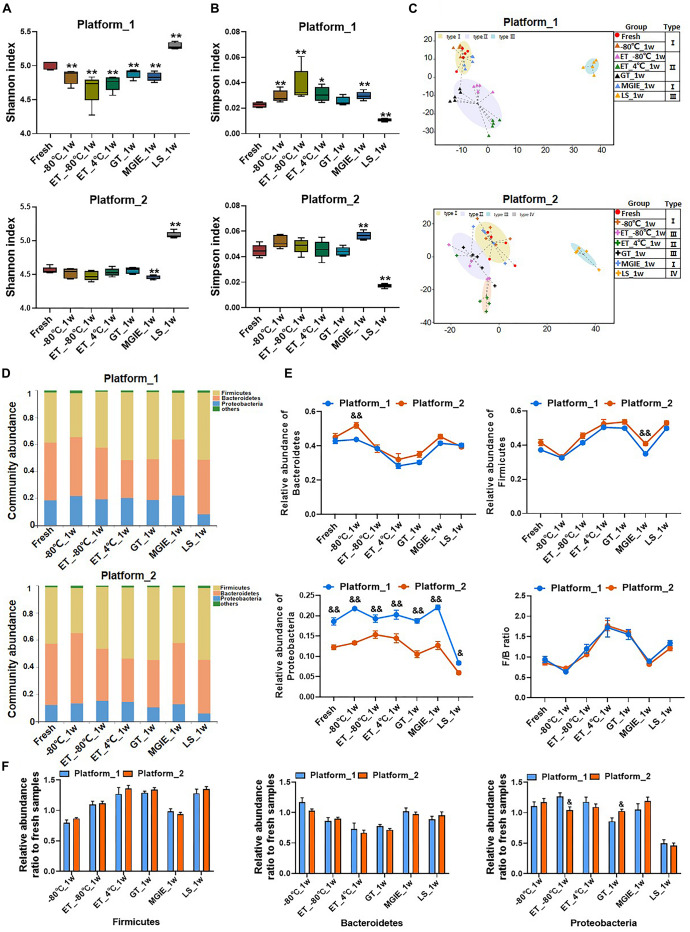
The impacts of different sequencing platforms on gut microbial profile. The community α diversity in platform_1 and platform_2 were analyzed by **(A)** Shannon diversity index and **(B)** Simpson diversity index. **(C)** The typing analysis on OTU level based on Jensen–Shannon Distance in platform_1 and platform_2. **(D)** Microbial communities under different storage conditions in platform 1 and platform_2. **(E)** The relative abundance of phyla Firmicutes, Bacteroidetes, Proteobacteria, and the ratio of Firmicutes to Bacteroidetes under different platforms. **(F)** The relative abundance ratio (stabilized samples to fresh samples) of the dominant phyla in different platforms. *n* = 5 per group. *p < 0.05, **p < 0.01, compared with fresh group; ^&^*p* < 0.05, ^&&^*p* < 0.01, compared with platform_1.

Given that most of the reported microbiome data in different studies are acquired on different sequencing platforms, the impacts of sequencing platforms on microbiome data are not very valued. To test whether different sequencing platforms will introduce biases in microbiome data, replicated DNA samples of same extraction from feces were shipped to two certified microbiome sequencing companies for sequence analysis on 16S rRNA gene according to their well-established protocols in our current study. The generated data were processed and analyzed by same researcher with same method. The comparisons between the data from two sequencing platforms included α and β diversities, intestinal typing, and community composition at phylum level. First, although our results indicated that the values of Shannon and Simpson indices from the two sequencing platforms slightly varied under the same storage condition, the general change trends of α diversity under different storage conditions was consistent between the two platforms (Figures 7A,B).

Then, the classification of dominant bacterial populations was performed with typing analysis based on Jensen-Shannon Distance. Samples from platform_1 were annotated into 3 types. Samples of fresh, −80°C and MGIE reagents were clustered as type I, and samples of ET_−80°C, ET_4°C, and GT reagents as type II, while only those in LS reagents were type III. Although samples from platform_2 were annotated into 4 types, the cluster patterns were similar with platform_1, except for a sub-clustering within type II, in which ET_4°C was differently classified with ET_−80°C and GT reagents (Figure 7C). The following community abundance analysis revealed the compositional structure of the dominant phyla between the same storage conditions including Firmicutes, Bacteroidetes, and Proteobacteria was comparable between the 2 sequencing platforms, even though the absolute values were of difference, especially in Proteobacteria (Figure 7D). Interestingly, similar to that of α diversity, the fluctuating trend of the dominant phyla under different storage conditions was consistent between the two platforms, which was clearly demonstrated by the overlapped *F*/*B* ratio under the 2 sequencing platforms (Figure 7E). Finally, to assess the alteration of the dominant phyla introduced by sequencing platforms more accurately, we compared the relative abundance ratio (stabilized samples/fresh samples) of Firmicutes, Bacteroidetes, and Proteobacteria between the 2 sequencing platforms. No significant variation in the abundance of the dominant bacteria between the two platforms, except for Proteobacteria at ET_−80°C and GT reagent (Figure 7F). In addition, we also performed a PCoA by integrating the samples from two platforms together, which showed a clear separation between the two platforms, however, the scatter pattern among all storage conditions was very similar within each platform, suggesting that the impacts of storage conditions could be similarly characterized by both platforms (Supplementary Figure S4).

Altogether, our results indicated that sequencing platforms introduced minimal alteration of bacterial abundance, moreover, the trend of fluctuation was highly consistent under different storage conditions on both sequencing platforms. Thus, the variations introduced by storage conditions surpassed that of sequencing platforms.

## Discussion

In the current study, we systemically evaluated the impacts of various fecal sample storage conditions, storage periods, and sequencing platforms on gut microbial profile based on 16S rRNA gene sequencing. Our results highlighted that gut microbiome profile varied to different extent under different sample storage conditions, which surpassed the impacts of storage periods and sequencing platforms.

There are huge amount of publications each year in the research on gut microbiota and human diseases (Li et al., 2017; Barcena et al., 2019; Canfora et al., 2019; McQuade et al., 2019), however, data of gut microbiome usually exhibited dramatic variations and inconsistency between studies (Walters et al., 2014). Generally, biases may be introduced during the whole experiment such as sample collection, transportation, storage, DNA extraction, and so on (Voigt et al., 2015; Costea et al., 2017; Al et al., 2018). Commercial stabilizer for preserving fecal samples at room temperatures are usually suggested due to the fact of sampling with no access to immediate freezing at −20°C or below or freezing transportation, especially in remote areas. Although the efficacy of some commercial stabilizers on maintaining the original status of gut microbiome has been compared, the uncertainty still exists because of the inconsistent observations among some reports (Hale et al., 2015; Sinha et al., 2016). Previous studies suggested that inter-individual variation from donors superseded that introduced by storage conditions (Guo et al., 2016; Bundgaard-Nielsen et al., 2018; Penington et al., 2018), however, the impacts of different storage conditions on microbiome profile are not well characterized. Therefore, we performed a multi-dimensional comparison among different storage conditions by using homogenized feces from rats to minimize the probable artifacts during sampling process. DNA extraction is a critical step for introducing variation to microbiome data if different protocols or extraction kits were used (Costea et al., 2017). To minimize the introduction of data variations by DNA extraction process, the genomic DNA of all samples was extracted by one experimenter with identical protocol in our current study. Although −80°C or GT reagents stored samples have been used as controls for evaluating the effectiveness of preservation methods in previous reports (Flores et al., 2015; Song et al., 2016; Han et al., 2018), the efficacy of these preservation conditions are still inconclusive because of the inconsistent results (Horng et al., 2018). Thus, we allocated a part of freshly collected fecal samples as the fresh control, in addition to −80°C. Stabilization of fecal samples with ethanol is an easy and economical way, and therefore different concentrations of ethanol were used for fecal sample preservation in previous reports including 100% ethanol for spider monkey (Hale et al., 2015), 95% ethanol for human or dog (Song et al., 2016), and 70% ethanol for canine fecal sample storage (Horng et al., 2018). However, inconsistent results were reported previously. For instance, Song et al. (2016) found that the preserving effect of 95% ethanol was comparable with that of FTA cards and OMNIgene Gut kit at ambient temperature, but strongly cautioned against the use of 70% ethanol, while Horng et al. (2018) demonstrated that samples stored in 70% ethanol showed the closest similarity with that of fresh samples. The inconsistency among these reports might be associated with differences in fecal sample donors or different definitions for the “Fresh” samples, for example, Horng et al. (2018) processed canine feces for DNA extraction after 2 h of temperature treatment, whereas Song et al. (2016) extracted DNA of human and dog feces on the day of donation. In our current study, to keep the “real” status of gut microbiome in fresh samples as most as possible, the fresh control samples were subjected for genomic DNA extraction within 15 min after collection. Compared to fresh control, storage at −80°C was ideal for maintaining the integrity of gut microbiome samples even though some variations were also present, while storing samples at −80°C with 70% ethanol showed advantages in long term storage. Notably, we found that samples at 4°C with 70% ethanol showed low similarity to that of fresh samples, which was inconsistent with observations from Horng et al. (2018).

Meanwhile, given its “excellent” performance in sample storage, GT reagent was usually considered to be an effective preservation methods and even used as control for evaluating the efficiency of other preservation methods (Choo et al., 2015; Song et al., 2016; Han et al., 2018). However, to the best of our knowledge, the similarity of microbiome profile between samples stored in GT reagents and fresh samples was unclear. Thus, in this study, we evaluated the performance of GT reagents, as well as another two commonly used commercial stabilizers, MGIE and LS, by comparing to fresh samples. Our results suggested that GT reagents might not be the most cost-effective reagents for fecal storage given its comparable performance in maintaining the original status of microbiome profile with other conditions such as MGIE, but relatively higher cost. On the contrary, the microbiome profile of samples in MGIE reagents was more similar with the fresh samples, whereas storage of samples in LS reagents introduced substantial variations in many aspects.

Since microbiota-derived metabolites like bile acids or SCFAs are critical for its functions (Sheng et al., 2017; Jena et al., 2018), the methodology-induced alteration of these functional bacteria should not be overlooked in microbiota study. In our current study, we specifically evaluated the abundance change of functional bacteria that are involved in SCFA-producing and/or bile acid metabolism. Our data suggested that different storage conditions could cause diversified fluctuations in the relative abundance of functional bacteria in a time-dependent or –independent way, and we also found that *Bacteroides* and *Lachnospiraceae_*NK4A136_group were relatively stable under these storage conditions. Moreover, the exact abundance of 6 functional bacteria in different storage conditions was qualified with samples stored for 6 months. We demonstrated that conditions of −80°C, ET_−80°C, MGIE or GT reagents introduced less variations, but higher variations in conditions of ET_4°C and LS reagents. Consistently, the comparable storage effects between GT and MGIE were previously reported (Han et al., 2018). These results suggested that the addition of preservation reagents may also influence the bacterial DNA extraction efficiency to different extent, and the variations of bacteria abundance. Although our current data could only provide limited evidence for evaluating the impacts of storage conditions on functional bacteria change, the results suggested that improper sample storage might give rise to bias in respect to the functional interpretation based on 16S rRNA gene sequencing. It is the first time, to the best of our knowledge, to evaluate the impacts of different storage conditions on these functional bacteria quantitatively. Our current results highlighted the importance of sample storage conditions, which may lead to false understanding based on the fluctuations of functional bacteria in disease.

Previous studies usually evaluated the impacts of short-term or long-term storage on gut microbial profile using samples stored ranging from 24 h to 8 weeks (Song et al., 2016; Al et al., 2018; Bundgaard-Nielsen et al., 2018). In our current study, the impacts of storage periods under different conditions were evaluated from 1 week to 6 months. Our results showed that the dominant OTUs abundance of samples stored at −80°C, ET_4°C and MGIE reagents resulted in time-dependent alterations. Meanwhile, in comparison with fresh controls, samples stored at −80°C, in either GT or LS reagents resulted in obvious variations in the dominant phyla time-dependently, while similar conclusion were not observed in the dominant genera. Meanwhile, our current study showed that most of the variable genera are belonging to Gram-positive. Our results are in line with previous observations that Gram-positive bacteria are more likely to be affected by DNA extraction due to their thicker cell wall (Maukonen et al., 2012; Costea et al., 2017). Generally, although some variations under different storage periods were observed, we found that variations between stabilized samples and fresh samples were larger than that of samples stored for different periods under the same storage condition, suggesting that variations caused by ways of storage condition were larger than that of storage period.

In addition, although variations in gut microbiome were mainly caused by the difference between individuals, sequencing is also a source for introducing variations in microbial data (Penington et al., 2018). Our current results showed that α diversity and the relative abundance at phylum level varied between the two tested platforms, nevertheless, the change trends between two platforms were still consistent among different storage conditions implying that biases introduced by sequencing platforms were less than that introduced by storage conditions. It should be noted that we cannot clearly determine the origin of the variations between two sequencing platforms because the whole sequencing process includes PCR amplification, library construction, sequencing depth and subsequent bioinformatics. Our current observation provided a general evaluation on impacts of sequencing platform as a whole, which suggested that if quantification of specific bacterium was needed, the variations introduced by the platform should also be taken into consideration.

## Conclusion

**FIGURE 8:**
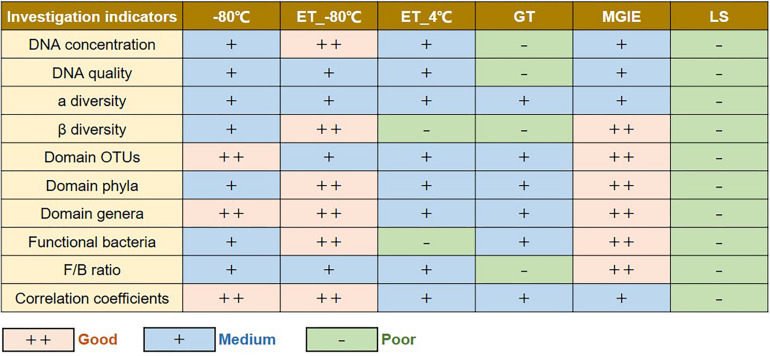
Summary table of the results.

In the current study, we performed a multi-dimensional evaluation on the variations introduced by types of storage conditions, preservation period, and sequencing platform on the basis of data acquired from 16S rRNA gene sequencing on rat fecal samples. As shown in Figure 8, our results suggested that, compared to fresh control, the impacts on genomic DNA quality and yields are LS > GT > MGIE > ET_4°C > −80°C > ET_−80°C in a time-independent way. Similarly, the impacts on α diversity are LS > −80°C > ET_−80°C > ET_4°C > MGIE > GT in a time-independent way. The impacts on β diversity are LS > ET_4°C > GT > −80°C > ET_−80°C > MGIE time-dependently. The impacts on abundance of functional bacteria are LS > ET_4°C > ET_−80°C > −80°C > GT > MGIE. In addition, the impacts of storage conditions > storage periods > sequencing platforms. Therefore, our current results underpin that the storage conditions for fecal samples should be consistent to minimize the deviation that would influence the final readouts during the microbiome study, while same storage periods and protocols are also suggested.

## Materials and Methods

### Animals

Male Wistar rats were provided by Shanghai Slack Laboratory Animal Co., Ltd. The animal experiments were conducted under the Guidelines for Animal Experiment of Shanghai University of Traditional Chinese Medicine and the protocol was approved by the institutional Animal Ethics Committee.

### Commercial Kits

Three commercial kits were used in the current study, including the Genotek OMNIgene⋅GUT OM-200 (DNA Genotek Inc., Canada), MGIEasy fecal sample collection kit (Shenzhen Huada Zhizao Technology Co., Ltd., China), and Longsee (Guangdong Nanxin Medical Technology Co., Ltd., China).

### Fecal Sample Collection and Processing

Fecal samples from 10 rats were quickly collected into sterile 50 ml tubes, joined into a single sample, and homogenized as much as possible. Next, these samples were aliquoted immediately, five technical replicates were performed under each condition, and aliquots were preserved using the following conditions: immediate freezing at −80°C with or without 70% ethanol (ET_−80°C), refrigerating at 4°C with 70% ethanol (ET_4°C), the use of OMNIgene⋅GUT (GT), MGIEasy (MGIE), and Longsee (LS) according to the manufacturer’s instructions and stored for 1 week, 2 weeks, and 6 months. All samples were shaken uniformly before stored at various conditions. Notably, according to the instructions, OMNIgene⋅GUT and MGIEasy samples were stored at room temperature, while Longsee samples were refrigerated at 4°C prior to DNA extraction. In addition to extracting DNA on the day of collection (fresh), extractions of other storage condition samples were conducted after 1 week, 2 weeks, and 6 months of storage, respectively.

### DNA Extraction

DNA extraction was performed using QIAamp Power Fecal DNA Kit (QIAGEN, Germany). For samples immersed in solution, aliquots were centrifuged at 13,000 *g* for 5 min, and the supernatant was discarded. The pellet was washed with PBS, and centrifuged again at 13,000 *g* for 10 min. Subsequent DNA extraction steps were performed according to the manufacturer’s instructions. In addition, DNA concentration and the A260/280 ratio were tested by Colibri Microvolume Spectrometer (TIRERTEK BERTHOLD, Germany).

### 16S rRNA Gene Sequencing

In our current study, DNA samples extracted at day 0, 1 week, 2 weeks, and 6 months of storage were applied to amplify the V3–V4 region of 16S rRNA gene using the universal primers 338F “ACTCCTACGGGAGGCAGCAG” and 806R “GGACTACHVGGGTWTCTAAT.” Library construction was conducted using TruSeqTM DNA Sample Prep Kit (Illumina, United States). Purified amplicons were pooled in equimolar and paired-end sequenced (2 × 300) on an Illumina MiSeq PE300 system (Illumina, United States), which is defined as platform_1 in the following experiments. Raw fastq files were demultiplexed, quality-filtered by Trimmomatic, and merged by FLASH with the following criteria: (i) The reads were truncated at any site receiving an average quality score <20 over a 50 bp sliding window. (ii) Primers were exactly matched allowing 2 nucleotide mismatching, and reads containing ambiguous bases were removed. (iii) Sequences whose overlap longer than 10 bp were merged according to their overlap sequence. The detailed information of sequencing was shown in Supplementary Table S1. Subsequent analysis was based on normalized data. Operational taxonomic units (OTUs) were clustered with 97% similarity cutoff using UPARSE (version 7.1^1^) and chimeric sequences were identified and removed using UCHIME. The taxonomy of each 16S rRNA gene sequence was analyzed by RDP Classifier algorithm^2^ against the Silva (SSU123) 16S rRNA database using confidence threshold of 70%.

In addition, to investigate impacts of different sequencing platforms on gut microbial profile, identical DNA samples from feces that were stored for 1 week were applied to amplify the V3–V4 region of 16S rRNA gene using the primers 338F “ACTCCTACGGGAGGCAGCAG” and 806R “GGACTACHVGGGTWTCTAAT,” both forward and reverse primers were tagged with Illumina adapter, pad and linker sequences. VeraSeq High-Fidelity DNA Polymerase (Enzymatics, United States) was used. PCR products were purified with AmpureXP beads and eluted in Elution buffer. Libraries were qualified with Agilent 2100 bioanalyzer (Agilent, United States), and the validated libraries were used for sequencing on Illumina HiSeq 2500, PE300 (Illumina, United States), and generating 2 × 300 bp paired-end reads, which is defined as platform_2 in subsequent analysis. In order to obtain more accurate and reliable results in subsequent bioinformatics analysis, the raw data was filtered (Fadrosh et al., 2014) and merged sequences whose overlap longer than 15 bp using FLASH (Magoc and Salzberg, 2011). The detailed tags statistics was shown in Supplementary Table S2. Subsequent analysis was based on normalized data. The tags were clustered to OTUs by scripts of software USEARCH (v7.0.1090) with a 97% threshold by using UPARSE (Edgar, 2013), and chimeras were filtered out by using UCHIME (v4.2.40). OTU representative sequences were taxonomically classified using RDP Classifier v.2.2 against the database Silva (SSU123) using confidence threshold of 70%. Raw fastq files were deposited to the Sequence Read Archive database under the accession number PRJNA561903.

### Quantitative RT-PCR

QRT–PCR was performed using SYBR Green (A25777, Thermo Fisher Scientific, United States), 96-well plates, and the CFX connect Real-Time System. Each well was loaded with a total of 20 μl containing 2 μl of genomic DNA of bacteria as template, 0.5 μl of target primers, 7.5 μl of water, and 10 μl of SYBR Select Master Mix. Hot-start PCR was performed for 40 cycles, with each cycle consisting of denaturation for 15 s at 94°C, annealing for 30 s at 60°C and elongation for 30 s at 72°C. Relative quantification was done using the 2^–ΔΔCT^ method. Values were normalized against universal primer of bacteria. Mean abundance of fresh samples was set as 100%. The primers used are shown in Supplementary Table S3.

### Statistical Analysis

The acquired raw data were normalized with the following criteria: (1) OTU with sequence numbers greater than or equal to 1 in at least 2 samples, that is OTU whose total sequence number is greater than or equal to 2 were included; (2) Normalized data were obtained after normalization to the minimum number of sample sequences. Alpha diversity was determined using Shannon’s diversity index, Simpson’s diversity index, Chao’s diversity index, and Shannon’s evenness index, that calculated by Mothur1.30.2^3^ (Schloss et al., 2009). The Principal Coordinate Analysis (PCoA) based on binary_jaccard, and Bray–Curtis were conducted to evaluate similarity of microbial community. The PCoA is calculated according to the OTU table after normalization, which is mapping based on the selected distance Matrix, and the difference between groups was analyzed by Adonis test. The classification of dominant bacterial populations under different storage conditions was studied mainly by typing analysis: based on the relative abundance of the bacteria at the OTU level, the Jensen-Shannon Distance (JSD) distance was calculated, and the PAM (Partitioning Around Medoids) cluster was calculated. Next, the clustering *K*-value is calculated by the Calinski–Harabasz index, and then principal coordinates analysis was used for visualization.

In our current study, statistical analyses were performed using the GraphPad PRISM version 8.0.1. Data obtained from experiments were shown as means or means ± SEM, differences between groups were calculated by Mann–Whitney *U* test or Kruskal–Wallis *H* test using SPSS 24.0 (IBM, SPSS, United States). In addition, the *p-*value was adjusted using Benjamini–Hochberg to control the multiple testing false discovery rate (FDR) in the analysis of the top 60 OTUs.

## Author’s Note

This manuscript has been released as a Pre-Print at bioRxiv (Ma et al., 2019).

## Data Availability Statement

The datasets generated for this study can be found in the PRJNA561903.

## Ethics Statement

The experiments were conducted under the Guidelines for Animal Experiment of Shanghai University of Traditional Chinese Medicine and the protocol was approved by the Institutional Animal Ethics Committee.

## Author Contributions

JM conducted the experiments, data analysis, and manuscript writing. LS helped in data analysis and manuscript writing. CX helped in fecal sample collection and storage. YH, YG, and NZ helped in data analysis. ML helped in false-discovery rate analysis. LC, GW, YL, JY, RH, BL, HQ, and JZ helped in animal experiment, sample collection and preparation. WJ participated in the design of this study. HL supervised the project and revised the manuscript.

## Conflict of Interest

The authors declare that the research was conducted in the absence of any commercial or financial relationships that could be construed as a potential conflict of interest.

## Footnotes

1 http://drive5.com/uparse/

2 http://rdp.cme.msu.edu/

3 https://www.mothur.org/wiki/Download_mothur
