# Supplementary material for: Variations of Gut Microbiome Profile Under Different Storage Conditions and Preservation Periods: A Multi-Dimensional Evaluation
Source: Front Microbiol. 2020 May 27;11:972. doi: 10.3389/fmicb.2020.00972 (PMC7267014; doi:10.3389/fmicb.2020.00972)
Supplement: Supplementary file 1 [file Data_Sheet_1.docx]

Supplementary Material


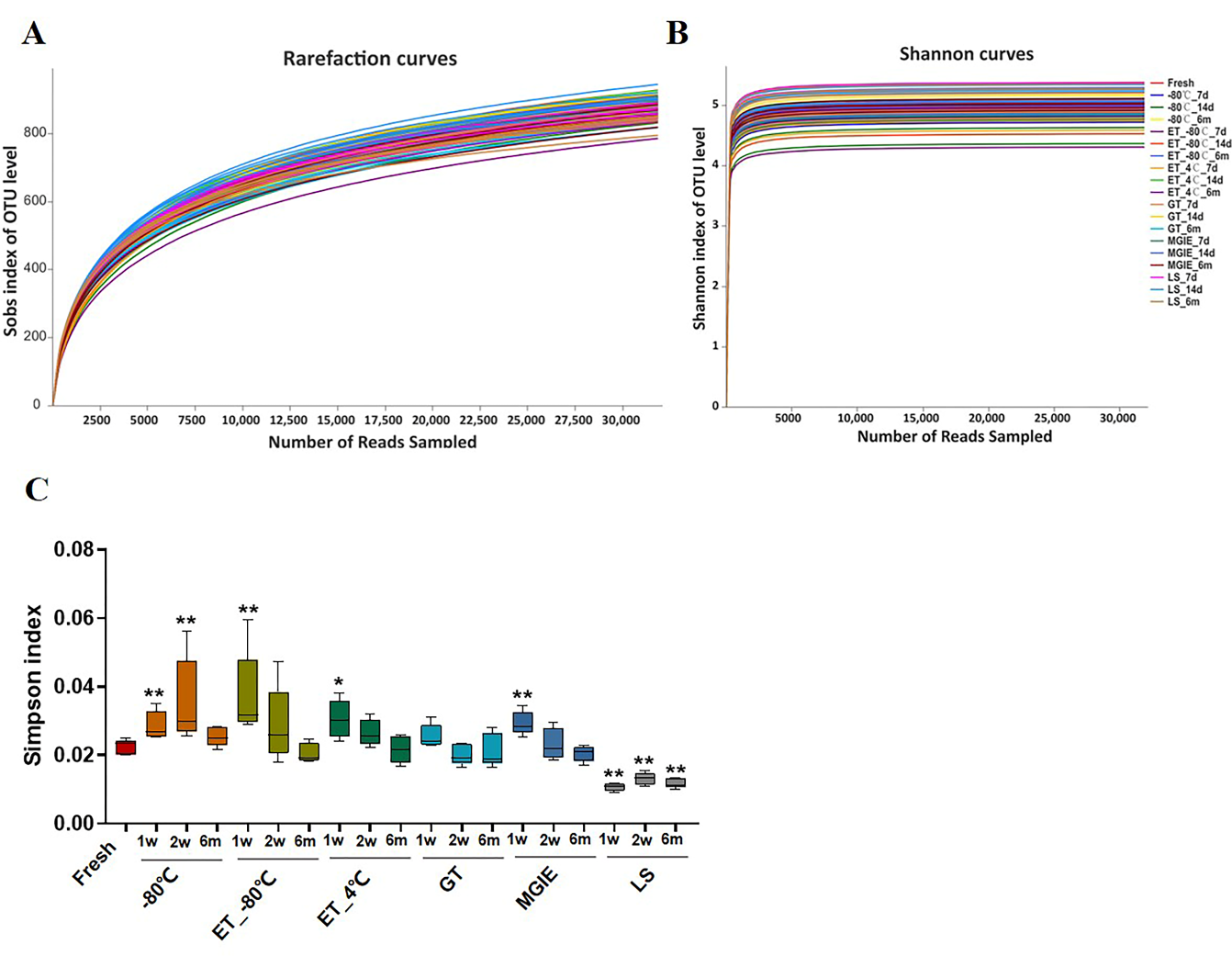


**Supplementary Figure 1.** The effect of preservation methods on α diversity.

**
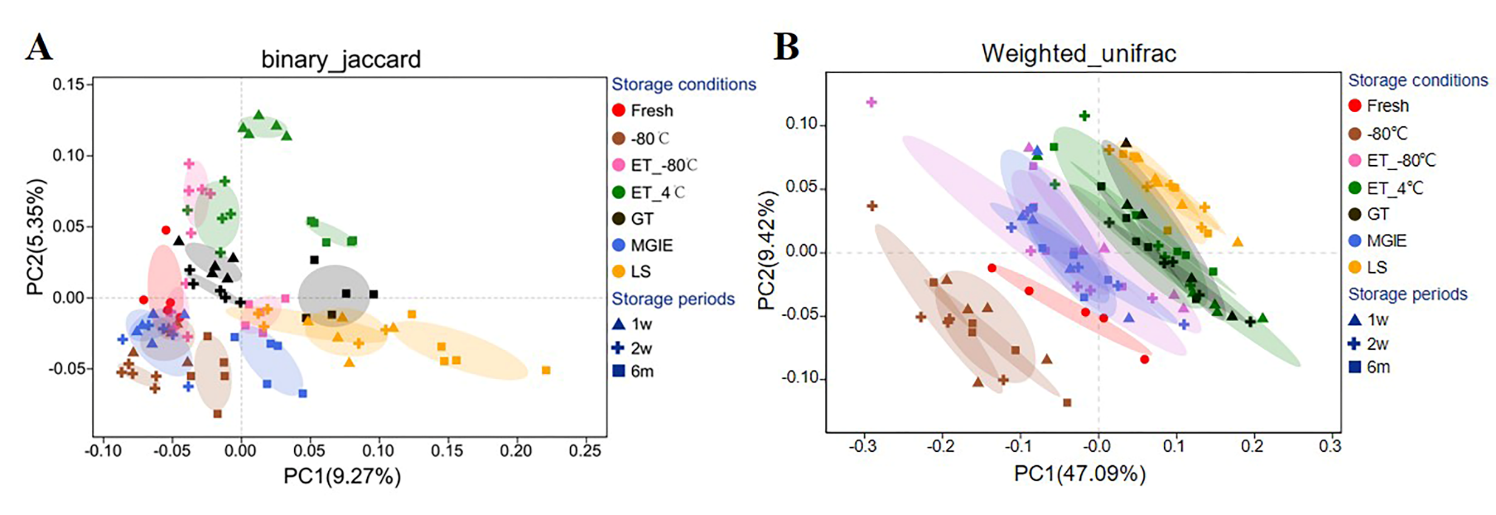
**

**Supplementary Figure 2.** The effect of preservation methods on community structure. PCoA analysis based on binary_jaccard (A) and weighted_unifrac (B).


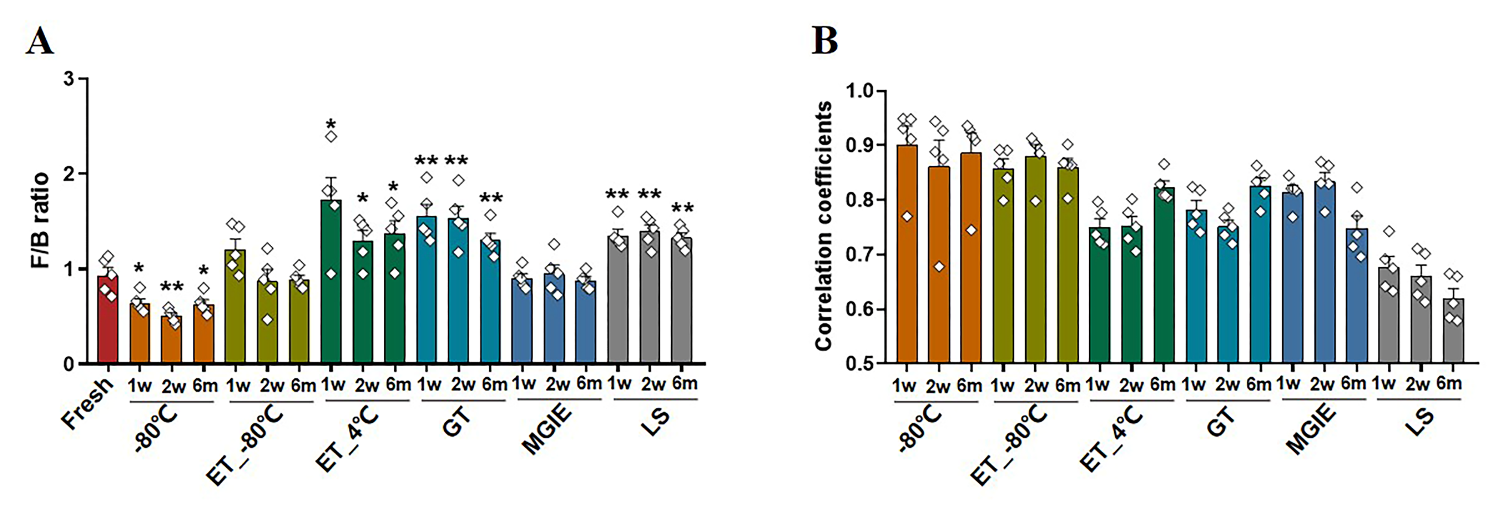


**Supplementary Figure 3.** The effect of preservation methods on the community composition. (A) The ratio of Firmicutes to Bacteroidetes under different storage conditions. (B) Spearman correlation coefficients between the stored samples and corresponding freshly extracted ones on genus level. *n* =5 per group. *p* < 0.05, ***p* < 0.01, compared with fresh group.


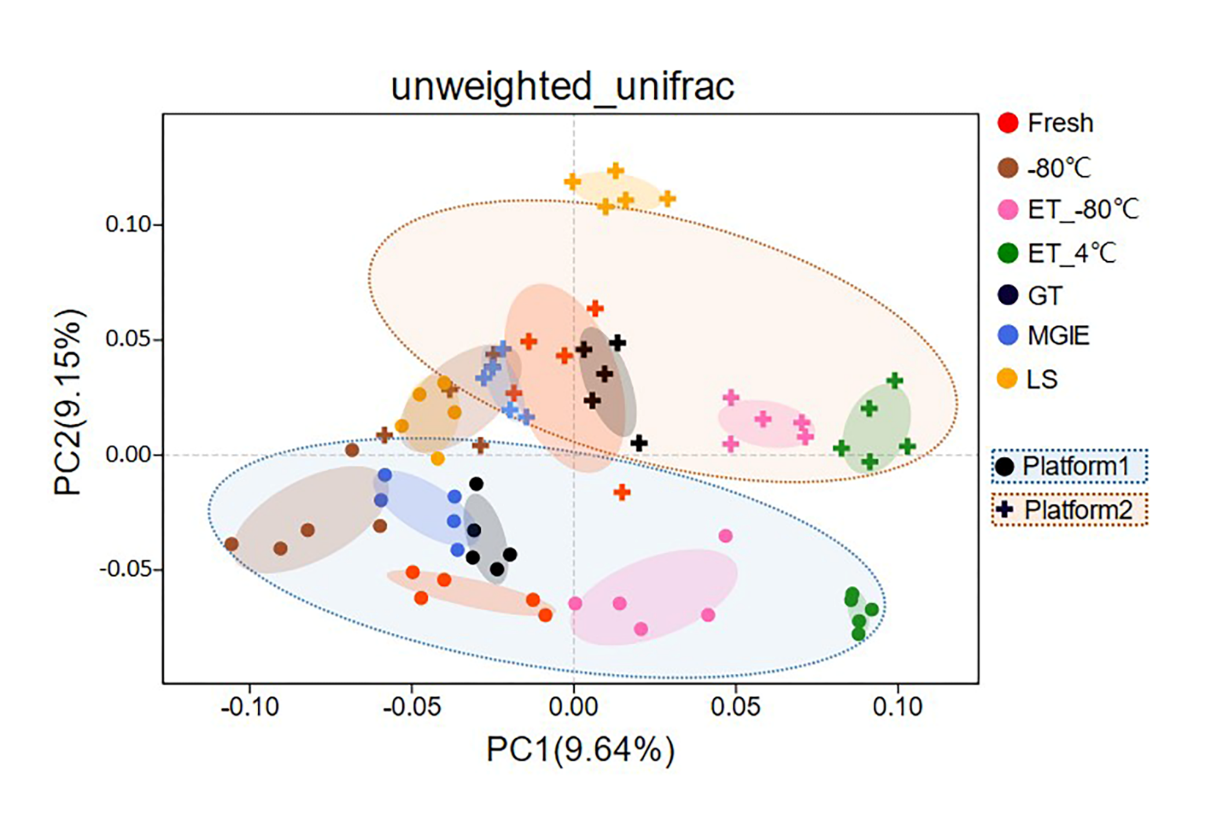


**Supplementary Figure 4.** The impacts of sequencing platforms and storage conditions on gut microbial profile.

**Supplementary Table 1**. Sequencing Information for Platform 1.

| Sample | Number of Sequences | Number of bases | Mean_length | Min_length | Max_length |
| --- | --- | --- | --- | --- | --- |
| Fresh_1 | 65839 | 28895298 | 438.8781421 | 318 | 453 |
| Fresh_2 | 60602 | 26641724 | 439.6179004 | 318 | 471 |
| Fresh_3 | 64813 | 28482557 | 439.45747 | 339 | 469 |
| Fresh_4 | 63875 | 27985596 | 438.1306614 | 291 | 454 |
| Fresh_5 | 60896 | 26706664 | 438.561876 | 358 | 453 |
| -80℃_7d_1 | 52302 | 23001959 | 439.7911935 | 341 | 476 |
| -80℃_7d_2 | 54041 | 23781016 | 440.0550693 | 331 | 454 |
| -80℃_7d_3 | 54984 | 24213889 | 440.380638 | 358 | 454 |
| -80℃_7d_4 | 57640 | 25372442 | 440.1880985 | 340 | 454 |
| -80℃_7d_5 | 54593 | 23998651 | 439.5920906 | 318 | 460 |
| -80℃_14d_1 | 58349 | 24493347 | 419.7732095 | 261 | 436 |
| -80℃_14d_2 | 45347 | 19065239 | 420.4299954 | 343 | 436 |
| -80℃_14d_3 | 52688 | 22101390 | 419.4767309 | 294 | 431 |
| -80℃_14d_4 | 54758 | 22983659 | 419.7315278 | 337 | 444 |
| -80℃_14d_5 | 56020 | 23523160 | 419.906462 | 253 | 433 |
| -80℃_6m_1 | 57874 | 24278490 | 419.5059958 | 327 | 475 |
| -80℃_6m_2 | 62878 | 26301996 | 418.3020452 | 262 | 444 |
| -80℃_6m_3 | 64515 | 27019696 | 418.8126172 | 212 | 498 |
| -80℃_6m_4 | 64015 | 26857177 | 419.5450598 | 270 | 433 |
| -80℃_6m_5 | 67205 | 28209353 | 419.7508072 | 206 | 433 |
| ET_-80℃_7d_1 | 53868 | 23575034 | 437.6445014 | 338 | 454 |
| ET_-80℃_7d_2 | 66894 | 29348529 | 438.7318594 | 305 | 459 |
| ET_-80℃_7d_3 | 55512 | 24321995 | 438.1394113 | 358 | 454 |
| ET_-80℃_7d_4 | 42167 | 18499179 | 438.7122394 | 358 | 451 |
| ET_-80℃_7d_5 | 51521 | 22542158 | 437.5333942 | 275 | 453 |
| ET_-80℃_14d_1 | 53232 | 22226767 | 417.5452172 | 337 | 432 |
| ET_-80℃_14d_2 | 49261 | 20663636 | 419.4725239 | 298 | 477 |
| ET_-80℃_14d_3 | 50963 | 21287857 | 417.7120068 | 317 | 501 |
| ET_-80℃_14d_4 | 54884 | 22865926 | 416.6228045 | 318 | 490 |
| ET_-80℃_14d_5 | 61572 | 25742917 | 418.0945397 | 245 | 444 |
| ET_-80℃_6m_1 | 59819 | 24978735 | 417.5719253 | 203 | 477 |
| ET_-80℃_6m_2 | 74769 | 31139247 | 416.4726959 | 203 | 433 |
| ET_-80℃_6m_3 | 59931 | 25003353 | 417.2023327 | 283 | 450 |
| ET_-80℃_6m_4 | 72843 | 30430295 | 417.7518087 | 203 | 447 |
| ET_-80℃_6m_5 | 72559 | 30275315 | 417.2509957 | 265 | 441 |
| ET_4℃_7d_1 | 64848 | 28341091 | 437.038783 | 270 | 465 |
| ET_4℃_7d_2 | 59535 | 26040229 | 437.3936172 | 340 | 464 |
| ET_4℃_7d_3 | 61173 | 26705874 | 436.5630916 | 292 | 452 |
| ET_4℃_7d_4 | 53040 | 23266781 | 438.6648002 | 372 | 453 |
| ET_4℃_7d_5 | 53879 | 23559898 | 437.2742256 | 322 | 453 |
| ET_4℃_14d_1 | 53902 | 22504242 | 417.5029127 | 266 | 433 |
| ET_4℃_14d_2 | 53074 | 22049924 | 415.4562309 | 294 | 444 |
| ET_4℃_14d_3 | 54639 | 22757351 | 416.5037977 | 278 | 433 |
| ET_4℃_14d_4 | 58404 | 24326379 | 416.5190569 | 277 | 433 |
| ET_4℃_14d_5 | 53476 | 22273723 | 416.5181203 | 220 | 436 |
| ET_4℃_6m_1 | 73226 | 30482739 | 416.2830006 | 281 | 433 |
| ET_4℃_6m_2 | 61814 | 25798436 | 417.3558741 | 277 | 446 |
| ET_4℃_6m_3 | 64377 | 26769053 | 415.8170309 | 278 | 439 |
| ET_4℃_6m_4 | 71786 | 29873481 | 416.1463377 | 233 | 464 |
| ET_4℃_6m_5 | 74528 | 31038760 | 416.4711249 | 203 | 472 |
| GT_7d_1 | 55325 | 24171971 | 436.9086489 | 358 | 455 |
| GT_7d_2 | 58240 | 25431732 | 436.6712225 | 269 | 453 |
| GT_7d_3 | 64572 | 28214483 | 436.9460912 | 273 | 454 |
| GT_7d_4 | 61963 | 26963224 | 435.1503962 | 342 | 452 |
| GT_7d_5 | 59506 | 25954088 | 436.1591772 | 360 | 461 |
| GT_14d_1 | 55601 | 23115353 | 415.7362817 | 361 | 510 |
| GT_14d_2 | 61771 | 25584973 | 414.1906882 | 247 | 510 |
| GT_14d_3 | 58778 | 24401402 | 415.1451564 | 261 | 472 |
| GT_14d_4 | 54372 | 22622155 | 416.0625874 | 250 | 434 |
| GT_14d_5 | 54470 | 22680393 | 416.3832018 | 263 | 492 |
| GT_6m_1 | 71086 | 29561508 | 415.8555552 | 280 | 459 |
| GT_6m_2 | 59434 | 24756645 | 416.5401117 | 299 | 527 |
| GT_6m_3 | 72122 | 29962896 | 415.4473808 | 304 | 482 |
| GT_6m_4 | 69817 | 29019661 | 415.653222 | 232 | 489 |
| GT_6m_5 | 69309 | 28815425 | 415.7530047 | 259 | 499 |
| MGIE_7d_1 | 52965 | 23239949 | 438.7793637 | 357 | 465 |
| MGIE_7d_2 | 52983 | 23256088 | 438.9349036 | 348 | 453 |
| MGIE_7d_3 | 57266 | 25069105 | 437.7659519 | 275 | 468 |
| MGIE_7d_4 | 56558 | 24768861 | 437.9373563 | 358 | 452 |
| MGIE_7d_5 | 62929 | 27593857 | 438.4919036 | 338 | 454 |
| MGIE_14d_1 | 59020 | 24545443 | 415.8834802 | 250 | 498 |
| MGIE_14d_2 | 57116 | 23804979 | 416.7830205 | 212 | 431 |
| MGIE_14d_3 | 50994 | 21289807 | 417.4963133 | 302 | 444 |
| MGIE_14d_4 | 57560 | 24068897 | 418.1531793 | 337 | 457 |
| MGIE_14d_5 | 51710 | 21595763 | 417.6322375 | 312 | 444 |
| MGIE_6m_1 | 71674 | 29903363 | 417.2135363 | 263 | 444 |
| MGIE_6m_2 | 74387 | 31013870 | 416.9259414 | 316 | 436 |
| MGIE_6m_3 | 67398 | 28078369 | 416.605374 | 203 | 444 |
| MGIE_6m_4 | 74573 | 31129734 | 417.4397436 | 293 | 524 |
| MGIE_6m_5 | 56598 | 23608150 | 417.1198629 | 239 | 465 |
| Ls_7d_1 | 50303 | 21952715 | 436.4096575 | 297 | 468 |
| Ls_7d_2 | 44992 | 19645337 | 436.6406695 | 340 | 454 |
| Ls_7d_3 | 52114 | 22679782 | 435.1955712 | 344 | 457 |
| Ls_7d_4 | 49592 | 21617450 | 435.9059929 | 329 | 454 |
| LS_7d_5 | 50095 | 21869528 | 436.5610939 | 359 | 503 |
| LS_14d_1 | 60982 | 25391254 | 416.3729297 | 203 | 477 |
| LS_14d_2 | 55575 | 23063161 | 414.9916509 | 277 | 438 |
| LS_14d_3 | 50467 | 20968986 | 415.4989597 | 276 | 433 |
| LS_14d_4 | 54868 | 22808588 | 415.6992783 | 272 | 510 |
| LS_14d_5 | 54678 | 22713734 | 415.4090128 | 225 | 447 |
| LS_6m_1 | 66689 | 27667611 | 414.8751818 | 304 | 433 |
| LS_6m_2 | 57173 | 23789219 | 416.0918441 | 226 | 516 |
| LS_6m_3 | 73924 | 30699617 | 415.2861993 | 277 | 478 |
| LS_6m_4 | 62359 | 25938659 | 415.9569429 | 274 | 444 |
| LS_6m_5 | 67356 | 28016307 | 415.9437467 | 239 | 520 |
| Total | 5645994 | 2395646299 | 40346.45748 | 27589 | 43704 |

**Supplementary Table 2**. Sequencing Information for Platform 2.

**Supplementary Table 3**. Primer sequences used in this study for microbial abundance.

| Bacteria | Forward/Reverse | Primer（5‘ to 3’） |
| --- | --- | --- |
| *Universal* | Forward | TCCTACGGGAGGCAGCAGT |
|  | Reverse | GACTACCAGGGTATCTAATCCTGTT |
| *Lactobacillus* | Forward | GGAAACAGATGCTAATACCG |
|  | Reverse | CACCGCTACACATGGAG |
| *Faecalibacterium prausnitzii* | Forward | GATGGCCTCGCGTCCGATTAG |
|  | Reverse | CCGAAGACCTTCTTCCTCC |
| *Enterococcus faecalis* | Forward | CCCTTATTGTTAGTTGCCATCATT |
|  | Reverse | ACTCGTTGTACTTCCCATTGT |
| *Clostridium Cluster* IV | Forward | TTAACACAATAAGTWATCCACCTGG |
|  | Reverse | ACCTTCCTCCGTTTTGTCAAC |
| *Bifidobacterium* | Forward | CTCCTGGAAACGGGTGG |
|  | Reverse | GGTGTTCTTCCCGATATCTACA |
| *Bifidobacteria* | Forward | GCGTGCTTAACACATGCAAGTC |
|  | Reverse | CACCCGTTTCCAGGAGCTATT |
